# Practical Observations on the Treatment of Interlaminary Opacities of the Cornea

**Published:** 1832-05

**Authors:** Wm. John Thomas


### INTERLAMINARY OPACITIES OF THE CORNEA.

### *Practical Observations on the Treatment of Interlaminary Opacities of the Cornea.* By Wm. John Thomas, M.R.C.S.

The opacities resulting from acute inflammations of the cornea are frequently so exceedingly troublesome to the practitioner, as to cause him considerable inconvenience, more especially in the treatment of severer descriptions of this disease.

The superficial films that tarnish the transparency of the eye are well known to be perfectly amenable to the art of surgery: they are cases of daily occurrence. Those denser opacities, which partake more of the character of Albugo, are however frequently abandoned by the practitioner as incurable; and if we take into consideration the great period of time which must elapse before any impression can be made upon these deposits, we may almost give up the cure as impracticable. I have frequently been compelled to turn away many cases of this description, from a conviction of the inutility of tormenting the unfortunate sufferers, where the probability of cure was so greatly against them.

In the course, however, of the observations which I have from time to time made upon the subject, I am inclined to consider the denser opacities more under the influence of the art of surgery than I had previously anticipated. We all know that after the acute inflammation has ceased, the deposition of lymph takes place. I do not mean, however, to state that the effusion does not occur during the inflammatory stage, but that the interlaminary deposits are the sequelæ of acute inflammation, and generally originate towards the termination of the inflammatory stage. Indeed, inflammations terminate by effusion, and the effusion is the consequence of the decrease of the arterial action. I have endeavoured to sketch out the progress of these effusions in a paper published in the number of this Journal for July 1830, on the Proximate Causes of Chronic Ophthalmia. I then traced the different steps taken by nature to terminate the inflammatory disorders of the eye, and paid particular attention to the pathology of the interlaminary depositions. The subject of the present paper will be the observations which I have to offer upon the surgical treatment of these opacities, and an examination into the comparative powers of nature and of art in their removal. I am aware that many of my readers may be somewhat sceptical upon the powers of nature to remove these interstitial bodies, because we daily observe numberless cases of this description which have existed for years without any progress being made towards their removal. On the other hand, we are continually treating cases of the filmary opacities which vanish under the influence of scientific applications. It may be demanded, how we can possibly institute a comparison between an active and a passive power with propriety: there certainly appears an absurdity upon the face of the proposition, which, nevertheless, paradoxical as it may seem, it shall be our business to resolve.

I before stated that cases occasionally present themselves of interlaminary effusions subsisting for years in the transparent cornea, and completely obscuring the patient's vision. These deposits, not being superficial, are frequently consigned over as incurable, and the patient deprived of the only possible hope of relief from the administration of the surgical art. I am persuaded, however, that where staphyloma does not exist, many cases of the more profound effusions may be treated with reasonable hopes of ultimate success; and that, too, upon the same physiological principles upon which we are daily proceeding in the removal of the more superficial films.

In the treatment of the filmary varities we use the ordinary stimuli, with an intention of exciting absorption: we may employ the gttæ Argenti Nitratis, for instance, and we generally push our remedies so far as to excite a degree of superficial vascularity, not amounting to inflammation: we allow the excited action to subside, and then revive it; thus, by local stimuli and constitutional administration, removing the superficial opacities. May not the deeper-seated deposits be removed upon the same principles? When part of the cornea is completely opaque, it certainly would be worth while to push our remedies further than usual. Let us suppose the following case: An interlaminary deposit occupying the centre of the cornea, and completely eclipsing the pupil, the circumference of the cornea transparent, through which the radiations of the iris are visible. We apply the usual remedies, the transparent cornea becomes congested, exceedingly irritable, and ultimately clouded. Now, in the generality of cases, I contend that the majority of practitioners would rest here, without proceeding further. We will not induce active inflammation again, (they say,) lest the circumference of the opacity should extend, and the case become worse when we leave it than when it first came under our care. I confess that this view of the subject is very prudent and plausible, and to me for some time set boundaries to my stimulating treatment; nor could I be persuaded to abandon this position, until a case occurred which in some degree altered my pathological opinions upon the subject. The case was as follows:

A. B., aged about nine years, was attacked with strumous inflammation of the cornea, which left a white interlaminary deposit behind. The effusion occupied rather more than the circumference of the pupil, which was perfectly eclipsed by the disease. When she came under my care, the eye was perfectly free from inflammation, and the opaque spot had existed in an indolent state for more than a year. A number of attempts had been made to remove it, but all remedies were unsuccessful.

Being convinced of the great improbability of causing the absorption of the opacity without injury to the organ, I declined taking charge of the case, and told the parent that there were no hopes of removing the opacity. The deformity, however, being so conspicuous, they solicited me to do what I could to ameliorate the child's condition. Having reflected, that probably there might be some chance of diminishing the circumference of the opacity, I directed the parents to bring the child frequently to me, and for nearly half a year I pushed the usual remedies so far as to excite considerable irritation, but not inflammation in the eye. Scarcely any perceptible effect was produced by these proceedings, and the treatment was suspended in consequence. I saw nothing of the child for about a month: she was then placed under my care for purulent ophthalmia, attended with acute inflammation. The attack was treated by the usual local remedies, and the patient was put under the influence of mercury, in order to subdue the interlaminary inflammation of the cornea which appeared to be progressing rapidly. After the subsidence of the inflammatory action, I was pleased to find that absorption had taken place in the opacity which had previously existed, portions of the old deposit had disappeared, a transverse bar of transparency extended across the opaque spot, and several radii of the same diaphanous nature were distinctly visible. Still, however, several opaque patches remained. The absorbing treatment was again had recourse to, and mercury and iodine were occasionally prescribed, as the symptoms appeared to indicate.

Absorption proceeded slowly, and was at length suspended altogether. About half a year afterwards, the girl was seized with another attack of inflammation: I determined to allow the disease to proceed for a day or two, and when the opaque patches were fairly surrounded with a field of vascularity, I arrested the morbid action by the administration of mercury, &c. As soon as ptyalism supervened, the arterial excitement subsided, and I had the satisfaction of finding several of the opaque patches disappearing. When the eye had attained a quiescent state, the patient could distinguish many objects when presented to her for inspection; the centre of the cornea for about a quarter of a line, presented a transparent appearance, through which the pupil could be detected. There still remained traces of the circumference of the opacity, and I have the case again under my care for acute inflammation, evidently the effort of nature to throw off the remaining patches. I have reasonable hopes that these will ultimately disappear, under the action of the exhalents and absorbents of the centre.

I have stated that I apprehend the exhalents have had their share in the removal of these opacities. If we consider the intimate and almost inseparable consolidation of the corneal laminæ in the adult, we may, indeed, wonder how any exhalation can take place between them; but I endeavoured to shew in my former paper, alluded to above, in what manner I conceived these deposits to take place between their almost inseparable cohesions. I endeavoured to establish, as a pathological principle, that these effusions are invariably preceded by interstitial absorption, and that I considered such absorption to be a specific operation of the presiding power. I apprehend that nature excavated these receptacles with her own hand to receive that overplus of fibrine projected by the vis à tergo into the capillary exhalents of the arteries.

It is well established, as a surgical axiom, that certain portions of the animal body undergo rapid absorption from the operation of permanent compression. In the proposition which I now predicate respecting the interlaminary excavations, we have this surgical axiom illustrated. That vessels permeate the several layers of the cornea is a fact sufficiently established, that these vessels in their natural state carry a fine pellucid fluid, is also perfectly indisputable; that in consequence of the extremely delicate nature of this fluid, the transparent vessels which contain it are capable of accommodating themselves to its peculiar stimulus, is another fact which will admit of no dispute. Now what I contend for is this: that when the tide of arterial blood rolls its grosser materials into these transparent vessels, a considerable distention must take place. If the layers of the cornea were connected by yielding communications with each other, all might proceed as usual, and no permanent opacities be the consequence: but this is not the case; for we find, upon anatomical examination, that the different layers are so intimately connected together as to be almost inseparable. What, then, are the consequences? By the powerful vis à tergo, the capillary arteries are filled, the circumvesting fabric will not accommodate itself to the accumulating influx, and, as a physical necessity, a direct compression must ensue. This pressure will cause the absorption of all the circumjacent tissues against which its operation is exerted. Thus we have the fact established, that these deposits are preceded by interlaminary absorption. Taking it for granted that this corrollary will not be disputed, I now come to the practical applicability of these principles to the treatment of the interstitial deposits.

I have before stated that these chronic opacities are so deeply seated as to be out of the reach of the ordinary treatment of the filmary varietes: we will, therefore, consider these extraneous bodies encysted. The cysts are the anterior and posterior proximate laminæ of the cornea. I can easily conceive, and my readers will readily grant, that the extraneous substance may become organized, and the relation which it will then maintain will be that of an additional intervening laminæ, with the simple exception of the peculiarity of opacity. Now, if nature, by her own unassisted powers, can remove a natural layer intimately connected with the cornea, may she not be expected to remove, by an exertion of a power previously exercised, a preternatural peculiarity less intimately consolidated than those particles of matter whose place it now occupies? No physiologist will dispute that parts of recent growth possess less vitality than those of longer duration. If this be granted, the principles of our future proceedings will be clearly evident: we must imitate nature.

Specific excitement must be administered, and the powers gradually increased, until not only the superficial, but also the deeper-seated, organs, are called into full vascularity. Let an artificial inflammation be raised, and conducted scientifically. Let the transparent vessels be again filled with red blood, and the hand of nature pointed by the fingers of art to the desirable object of interlaminary excavation. What parts will be the most likely to be attacked, the dense and compact layers of the cornea? (the circumference of which is presumed to be transparent:) Certainly not; but that portion which possesses the minimum of vitality will be first assailed. Now, this is the precise spot which it is our duty and desire to demolish. The superficial vessels are first to be excited, and their excitement is to be carried on until the more profound arteries partake of the congestion. The deeper-seated vascularity will produce the distention, which will be followed by the direct pressure, and this pressure will cause the excavation of the surrounding particles which connect the foreign substance with the natural layers. Under these circumstances, it is clearly evident that, if the removal of the chronic opacity be not incompatible with the safety of the eye, it must undergo absorption. The patient will thus have an opportunity of regaining his vision by the removal of the opaque spot; and it is for his professional adviser to determine, upon due and cautious consideration of the extent of the disease and nature of the case, whether it would be prudent to undertake the difficult task before him.

I do not wish to intimate that we are to anticipate a complete dissolution of the opacity from one trial alone. The facts of the case which I have selected to illustrate the preceding propositions will not bear us out in such expectation. It may be necessary to repeat the process of inflammation several times; and in this we observe and imitate the proceedings of nature.

I am well aware that few patients will be found to submit to this severe ordeal, and probably still fewer practitioners will be bold enough to prescribe it: yet we are to remember that, if there be a rational probability that the posterior lamina of the cornea is transparent, and that the constitution of the individual will bear the treatment necessary, it is our duty to give the patient the only chance which art can afford him of restoration to sight by the removal of the deformity. The operation for the artificial pupil would be much more agreeable to both parties; but when we consider the great deformity which these opacities produce, and when the sex and state in society of the patient are taken into consideration, I think that individuals may be found who will gladly persevere, through time and tribulation, in the attainment of an object of such paramount importance.

*Liverpool; April* 7, 1832.

